# Household Contamination with Methamphetamine: Knowledge and Uncertainties

**DOI:** 10.3390/ijerph16234676

**Published:** 2019-11-23

**Authors:** Emma J. Kuhn, G. Stewart Walker, Harriet Whiley, Jackie Wright, Kirstin E. Ross

**Affiliations:** 1 College of Science and Engineering, Flinders University, Adelaide, SA 5001, Australia; stewart.walker@flinders.edu.au (G.S.W.); Harriet.whiley@flinders.edu.au (H.W.); jackie.wright@flinders.edu.au (J.W.); kirstin.ross@flinders.edu.au (K.E.R.); 2 Environmental Risk Sciences, Sydney, NSW 2118, Australia

**Keywords:** methamphetamine, guidelines, health risk, third-hand exposure, house, home

## Abstract

Contamination of residential homes with methamphetamine is an emerging issue of significant concern to public health. Cooking or smoking methamphetamine in a residential property contaminates the house, furnishings and personal possessions within it, with subsequent exposure through ingestion, dermal absorption and/or inhalation causing adverse health effects. Current guidelines identifying levels of methamphetamine contamination that require remediation vary between countries. There is also no international standard protocol for measuring levels of contamination and research has shown that different materials give rise to different recovery rates of methamphetamine. There are a number of currently used remediation methods; however, they have varying levels of success with limited studies comparing their long-term efficacies. Most importantly, there are few guidelines available that are based on a transparent, health risk-based approach, and there are many uncertainties on exposures and health effects, making it difficult to ensure the health of people residing in homes that have been used to cook or smoke methamphetamine are sufficiently protected. This manuscript presents the current state of knowledge regarding the contamination of residential homes with methamphetamine and identifies the current gaps in knowledge and priority areas for future research. The current regulatory approach to public health protection associated with exposure to residential premises contaminated with methamphetamine in Australia, New Zealand and the USA is also discussed.

## 1. Introduction

Amphetamine type stimulants (ATS) are a group of synthetic drugs that include amphetamine, methamphetamine (Ice) and 3,4-methylenedioxymethamphetamine (MDMA, ecstasy). Methamphetamine is available in four forms: a sticky, waxy base; ground, whitish powder; pills; and crystalline shards [1]. While all forms are highly addictive, the crystalline form, also known as ‘ice,’ is the purest form of the drug and therefore has increased potency [2]. Methamphetamine is commonly manufactured in clandestine drug laboratories either for personal use or for the illegal drug trade [1]. During manufacturing processes aerosols are released into the surrounding atmosphere [3,4,5]. The airborne residues and volatile organic compounds (VOCs) can settle on surfaces, such as walls, benchtops and flooring, where they can then be transported throughout the premises via air or dermal transfer. Larger scale operations, and a number of smaller personal cooking operations have permanent manufacturing locations that may house large volumes of chemicals and equipment, while the smaller setups or “box labs” are designed for short term manufacturing that can be well hidden and packed up quickly for relocation [6]. Clandestine laboratories can be setup in residential properties, hotel rooms, warehouses and spaces as small as a car trunk [7,8]. To date, most research has concentrated on residues from methamphetamine manufacturing. However, smoking methamphetamine has also been shown to produce residue contamination [4], though at much lower levels than measured during controlled cooks. Smoking, however, may be repeated on many occasions, resulting in higher levels of contamination.

A common scenario for contaminated properties is that the methamphetamine users or manufacturers vacate the premises and the residues remain within the house. Often, the new residents are unaware of the previous activities. These new occupants are exposed to the residual methamphetamine, and often other chemicals associated with production, resulting in adverse health effects [5,7]. Wright et al. [9] found these adverse health effects are diverse, and can include behavioural changes, respiratory illnesses and skin related responses. Age and the activities of the person exposed can influence the severity of the effects [9]. Symptoms can include irritability, anxiety, sleeplessness, weight loss, a persistent cough, dizziness, difficulty in breathing, nausea and throat, eye and skin irritations [9,10]. Occupants may not associate their symptoms with contamination in their home, and often investigate other environmental factors, such as mould, pests, vermin, allergies (e.g., hay fever) and contaminated water [11].

This paper provides a current state of knowledge regarding methamphetamine contamination and exposures that are of relevance for residential properties. The current methamphetamine remediation guidelines and regulations for Australia, New Zealand and the United States (US) are also discussed. The key gaps in knowledge and priorities research areas are identified and described.

## 2. Public Health Regulation

Guidelines generally cover the amount of contamination of methamphetamine and/or amphetamine that should trigger remediation and are also used to evaluate the success of the remediation itself. Once a property has been deemed contaminated, quantitative testing of methamphetamine is necessary to determine whether remediation is required. Post remediation testing is also required to ensure methamphetamine as well as the precursors and by-products of manufacture are properly removed, and no chemicals have desorbed from porous surfaces [12]. However, the precursors and by-products are generally not tested for, and the reduction in methamphetamine concentration is generally assumed to indicate a reduction in the other potential contaminants [13]. The four main methods used for production are the iodine/hypophosphorus (or hypo) method, the Birch reduction (or Nazi) method, the red phosphorus method and the phenyl-2-propanone (P2P) method [7]. While methamphetamine is the desired end product, many other chemicals are released during manufacture, and the aerosols and other by-products released during production are dependent on the manufacturing method [14]. Additionally, the reduction in methamphetamine concentration could be due to a conversion to different chemicals that do not test positive in methamphetamine testing but may be harmful.

Health risk-based guidelines are generally quantified based on observed physiological and behavioural parameters, uncertainty and safety factors. A reference dose (RfD), is assumed to be protective and adverse health effects for all members of the population [15]. There are currently uncertainties in both estimating and understanding the exposure dose, and therefore, developing appropriate RfD and toxicological guidelines.

Currently, the maximum acceptable amount of methamphetamine present on a surface differs between countries, and in the case of the US, between states. In addition to this variation, there is also often an absence of guidelines, standards and regulations for methamphetamine remediation. To illustrate, guidelines from Australia, New Zealand and the US, and some of the historical and contextual aspects associated with these guidelines, are presented below.

### 2.1. Australia

Due to the prevalence of clandestine laboratories discovered in Australia [16], the National Clandestine Drug Laboratory Remediation Guidelines were developed and released in 2011. This included quantitative health risk-based remediation guidelines for methamphetamine, and other contamination in residential and commercial/industrial settings. For residential homes the methamphetamine guideline is 0.5 µg/100 cm^2^ (Table 1). The report presents the four phases of site remediation, from the initial assessment to the decontamination of the property and it being verified and documented in a validation report [16]. While these guidelines provide valuable information for all stakeholders, especially homeowners and professional remediators, they have not yet been cited in legislation and are therefore voluntary and not enforceable. All six states and two territories in Australia, except Queensland and the Northern Territory, have developed their own clandestine decontamination guidelines, all of which reference the National Guidelines.

In addition to the National Clandestine Drug Laboratory Remediation Guidelines, enHealth (the federal body that provides environmental health policy advice in Australia) recently produced the enHealth Guidance on: Clandestine Drug Laboratories and Public Health Risks [17], a position paper that informs the states about the current scientific evidence indicates in relation to this matter. Unfortunately, while enHealth highlights the public health importance of this issue, none of the individual states have made a strong stand or implemented legislation to support the enHealth statement.

Clandestine laboratories that have been seized and investigated by police are generally deemed contaminated, and a notice requiring assessment and remediation will be put on the property by the local council [16]. However, how clandestine laboratories and contaminated properties are handled in Australia varies and is dependent on the legislation in each of the states. Environmental health officers from local councils can implement relevant state legislation by assigning a notice to the property for being a suspected clandestine laboratory and a concern for public health [18,19,20,21,22,23]. While this notice is to highlight suspected contamination and prevent human inhabitants from living at the property prior to remediation, there is currently no regulatory body nor legislation to ensure compliance. Currently, Western Australia have nominated local government or the Department of Communities for Public Housing to regulate the decontamination [24].

However, if a homeowner suspects methamphetamine contamination, they undertake their own assessment and choose whether or not to remediate, as there is currently no protocol to formally notify the local council, and there is no regulatory requirement to remediate the property.

There has been increased demand for commercial cleaning companies that can remediate residential properties contaminated with methamphetamine. However, just as there is no regulatory body to ensure notice compliance, there are also no regulations for remediation companies to adhere to. This has created confusion and uncertainty for companies with regard to legal liability and ethical obligations with respect to residential property remediation. Notably, there have recently been media reports of a testing and remediation company allegedly exaggerating the levels of contamination to elicit a contract for remediation [25]. The implementation of standardised testing and remediation, with regulations and clear legal responsibilities, is clearly required in Australia.

### 2.2. New Zealand

In 2010, the Ministry of Health released a recommended methamphetamine decontamination limit of 0.5 µg/100 cm^2^ [51] This voluntary guideline provided information that sought to maintain consistency for occupational hygienists, law enforcement, local councils and homeowners. During this time, many public housing residents were evicted from their homes in the public housing sector as there was a zero tolerance policy put in place for methamphetamine contamination. This policy did not consider the quantification of the residues, when the property was contaminated and there was no differentiation between use and manufacturing of methamphetamine [52]. In the interests of public health, local councils have the capacity to enforce testing and remediation, and record all information on the Project Information Memorandum (PIM) or Land Information Memorandum (LIM) [53,54].

In 2016, the Institute of Environmental Science and Research Limited (ESR) assessed the decontamination level for methamphetamine laboratories and released a report with a proposed limit of 1.5 µg/100 cm^2^ and 2.0 µg/100 cm^2^ for properties with and without carpet respectively [13]. These limits were for properties that were exposed to methamphetamine use, while the decontamination limit for remediating clandestine laboratories was maintained at 0.5 µg/100 cm^2^. Standards New Zealand coordinated a committee that consisted of a range of different stakeholders, including local councils and government, analytical testing companies, real estate representatives and commercial cleaning companies. In 2017, a New Zealand Standard (NZS 8510:2017) was released which set the decontamination limit to 1.5 µg/100 cm^2^ for high use areas and 3.8 µg/100 cm^2^ for areas with limited use [27] (Table 1). There was no distinction made between methamphetamine use or production within the report; this is due to the difficulty in distinguishing between use and manufacture in properties that no longer have any evidence of manufacture.

ESR released an internal report in 2018 that was a re-assessment of the existing decontamination limits, and was compiled using New Zealand housing data and the New Zealand Standard [55]. Subsequently in 2018, a report was released from the Office of the Prime Minister’s Chief Science Advisor (PMCSA), “Methamphetamine contamination in residential properties: Exposures, risk levels, and interpretation of standards.” The main proposal from the PMCSA report was to increase tenfold, the decontamination limit from the NZS 8510:2017 standard of 1.5 µg/100 cm^2^ to 15 µg/100 cm^2^ for properties only involving methamphetamine use [56]. However, if manufacturing was suspected, it was recommended that remediation should remain at 1.5 µg/100 cm^2^ [56].

The PMCSA report suggested the presence of residual precursor chemicals as the difference between manufacturing and use, and consequently, ESR advised limits of 0.5 µg/100 cm^2^ and 2.0 µg/100 cm^2^ respectively [13]. The assumption was that methamphetamine alone is a low risk to human health, and the variance accounts for the other chemicals involved in manufacturing. According to the PMCSA report, these precursor chemical residues are not commonly found in New Zealand, a statement that originates from an internal report released by ESR [56]. This internal report is often cited and referenced; however, the public can only view a heavily redacted version made available through New Zealand’s Official Information Act 1982. The PMCSA report has instigated major changes in the public housing sector, yet the public are unable to view or verify the research that is used to support these changes. This lack of transparency is concerning, as at present there is significant confusion and misinformation surrounding methamphetamine contamination and its remediation and government reports should address, rather than increase, this confusion.

There are a number of aspects of the PMCSA report that further add to the confusion about methamphetamine contamination. There is acknowledgement throughout the PMCSA report of the toxicity assessments available from California, where a reference dose (RfD) is determined, and Colorado where a standard is determined [57,58,59]. However, the US reports are not considered in detail and are, to an extent, dismissed, as use only is considered to cause negligible contamination. The report states that testing or remediation is not necessary for levels of less than 15 µg methamphetamine per 100 cm^2^. However, it is also stated that low methamphetamine levels do not eliminate manufacturing as the source of contamination. It was also noted that testing at low levels should only be undertaken if there is evidence to suspect manufacturing. This is concerning, as the contamination level can only be determined if testing is performed. This approach requires that drug manufacturing paraphernalia, or specific visual staining, must be present as evidence to suspect manufacturing. However, it has already been well established that it is not always obvious whether a property is a former clandestine laboratory [9,60,61,62]. While the PMCSA report stated that forensic experts found over 30 µg/100 cm^2^ can suggest methamphetamine production [56], levels below this does not preclude manufacture and without quantitative testing, the contamination level cannot be known.

The PMCSA report states that the New Zealand Standard (NZS 8510:2017) has not been cited in legislation, and therefore cannot be legally enforced [56]. However, notably, since the PMCSA report has been released, Housing New Zealand (the government organization that controls public housing in New Zealand) has raised the decontamination limits to 15 µg/100 cm^2^ [52], despite the PMCSA report being only a recommendation and in contrast with the standard (NZS 8510:2017). According to the Tenancy Tribunal [63], both the New Zealand Standard and the PMCSA limits are acceptable as the Residential Tenancies Act currently does not state a specific limit. This adds to the confusion and uncertainty surrounding methamphetamine remediation.

The PMCSA report indicates that it is preferable that residents are exposed to “low levels” (by which they mean <15 µg/100 cm^2^) methamphetamine contamination in comparison to unstable public housing situations [56]. Many residents from social housing are disadvantaged and from lower socio-economic demographics [64]. The social determinants of health have clearly demonstrated that lower socioeconomic groups suffer significantly poorer health outcomes compared with their richer counterparts [65]. The people in this demographic are less able to make their own decisions about whether to live in a contaminated environment as it is unlikely that they can afford the costs associated with testing and remediation. The acceptability of exposing residents to “low” levels of methamphetamine contamination to avoid disrupting their housing arrangements [9,56] is debatable and is a decision that needs to be made in light of all available evidence. It is therefore unacceptable that much of the supporting report to the PMCSA report is redacted. The safety factors and buffers that are used for California’s RfD and Colorado’s standard are also mentioned a number of times in the PMCSA report. These safety factors are put in place for the purpose of addressing uncertainty in knowledge and protecting public health. Therefore, it is concerning that there is no regard to the criteria presented in the New Zealand Standard (NZS 8510:2017), and instead, the report states that there is no health concern above 1.5 µg/100 cm^2^, as there is already a built-in conservative safety buffer, and has therefore deemed that 15 µg/100 cm^2^ can actually be considered safe [56].

The reports released from Ministry of Health, ESR and Standards New Zealand are voluminous (176, 55 and 61 pages each respectively) The conflicting reports are likely to cause confusion and uncertainty and could potentially increase mistrust in government and standardisation bodies that exist to provide support and structure for the general public. There is also the possibility that this could potentially lead to legal class action in the future (as happened with asbestos and glyphosate, for example).

### 2.3. United States

In the US, the ‘Methamphetamine Remediation Research Act of 2007’ was proposed and accepted to enable Federal research for the development of methamphetamine remediation guidelines [66]. This Federal funding enabled the Environmental Protection Agency (EPA) to develop remediation and sampling guidelines [67]. While the EPA guidelines are from a Federal agency, they are still voluntary and are not enforced by any legislation. The guidelines and regulations were determined individually by each state which has created inconsistencies across the US. It has been suggested that several US states based their methamphetamine clean-up standard on the original target value (0.1 µg/100 cm^2^) promoted by the State of Washington. The Washington standard was not developed using health risk assessment methodology; it was simply based on the analytical detection limit for the drug (personal communication, Anonymous, 2019). Other states have adopted this limit because for many years it was the only standard available ((personal communication, Anonymous, 2019). ibid.). Therefore, this has resulted in guidelines that range from 0.05 to 1.5 µg/100 cm^2^ (Table 1), regardless of the cause of contamination. Few states have considered contamination from both use and manufacture; Minnesota has a limit of 0.1 µg/100 cm^2^ for former labs and Colorado allows up to 4 µg/100 cm^2^ in limited exposure areas, using a health risk-based approach. States such as Utah, Montana and Oklahoma include various disclosure laws which require the contamination at a property to be disclosed at sale. While a number of individual states have established guidelines, many of which are in state legislation and regulations, there are a significant number of individual states that have not set any remediation standards or regulations for methamphetamine contamination.

## 3. Assessing Household Contamination

It has been shown that methamphetamine residues remain on surfaces, upholstery and flooring post-cooking and post-smoking [4,68,69]. These surfaces may also desorb methamphetamine over time, and when these residues are disturbed, they become airborne within the environment. Generally airborne emissions are higher closer to the original contamination and there is methamphetamine that can be re-released into the air days or years after deposition [12,69]. During simulated smoking and controlled cooks of methamphetamine, airborne particles were found to vary in size from <1.0 µm to >2.5 µm [70]. These particles were easily transported and did not require any physical activity in the area to detect residues in excess of the 0.5 µg/100 cm^2^ decontamination limit [4,70]. For the smoking of methamphetamine, it has been estimated that approximately 67% is inhaled, which leaves 33% to be deposited on the surrounding environment [71]. Van Dyke et al. [70] demonstrated the mobility of methamphetamine through a controlled cook; not only did pre-cook wipe samples have lower contamination than post cook wipes, but increased levels were found in six different locations within the house. In study from Martyny et al. [14], it was suggested that higher quantities of methamphetamine residue could travel over 4 metres, depending on the manufacturing method used. These studies illustrate the variation in an area as a result of transport of methamphetamine in the time period just after manufacture. A study from Wright et al. [69] subsequently demonstrated the transfer of residue continues to occur long after production which results in contamination of new items brought into the property.

Research into contamination levels on different surfaces has shown that the recovery from different surfaces is highly variable. For example, recovery of methamphetamine from granite, limestone and marble, 2.5 h post contamination was 28% and 27% and 6% respectively [72]. Recovery after 48 h had decreased to 19%, 8% and 0.7% respectively [72]. Abdullah and Miskelly [73] investigated the volatilisation of methamphetamine from open and covered surfaces (covered with a glass plate). The percentage of methamphetamine detected on the glass plate ranged from 9.3% to 21.7% of the free base, and 5.3% to 24.2% for the hydrochloride methamphetamine, which is an indication of particles becoming airborne [73]. These studies illustrate the challenges that can arise from contamination assessment using wipe sampling, as methamphetamine can become embedded into household surface materials and therefore may not provide a complete picture. A study from Serrano et al. [74] found that drywall could be decontaminated by 81% after three surface washes. However, it was also found that using a methanol wipe on drywall, only 37% of the methamphetamine was recovered, yet 58% still remained ingrained in the surface [74]. This finding was supported in a study from Wright et al. [69], which found significant penetration of methamphetamine through the paper outer layer of drywall into the calcium sulphate centre. Contamination was also present on the internal paper layer and the timber frame. Methamphetamine from this property was detected on pre-existing and introduced household surfaces over five years from the suspected manufacture of the drug [69]. The highest readings were found in the vacuum cleaner bag (97 µg/g) and the plastic vertical blinds (15–150 µg/g) in the room suspected to be used for synthesis [69]. Other studies have shown higher methamphetamine levels on post wipe samples, and interestingly, more methamphetamine was recovered from painted drywall than unpainted drywall at a low pH [75].

Research has considered the hazards to young crawling or walking toddlers, in which contact points accessible to children were analysed. The studies found that one of three contact points exceeded a 0.5 µg/100 cm^2^ decontamination limit which was verified by the 0.54–29 µg/100 cm^2^ result of the personal wipes collected from the crawling simulation [70,76]. The samples taken from fabrics associated with infants, were seen to have a higher average equilibrium partition coefficient for methamphetamine at 60% relative humidity [77]. This is a cause for concern, as infants tend to have hand to mouth behaviours and will mouth and touch toys, clothing, blankets and other items within reach. Clothing, fabric and upholstery items were easily contaminated; however, it was found that using a standard washing machine, over 91% of the methamphetamine was removed [74]. Wipe and post wipe sampling of fabric such as carpet and linoleum provided an example of the high levels that can be retained. Artificial leather had the lowest extraction efficiency when compared with acrylonitrile butadiene styrene plastic, silicon and laminate [5,68].

There has been research into the absorption of methamphetamine in artificial and natural skin oils, and sebum oil without fatty acids. This showed evidence that methamphetamine absorption was higher in the natural and artificial skin oils opposed to the oil without fatty acids, especially at increased relative humidity [78]. Career opportunities or job retention through compulsory drug testing could be affected, as evidence of skin penetration and positive hair analysis was reported in a case study of race horses in a methamphetamine-contaminated float for six hours which resulted in a positive drug test [79].

## 4. Remediation of Methamphetamine Contamination

The current remediation techniques for methamphetamine contamination are mostly adapted methodologies from existing cleaning protocols and are not necessarily evidence based. Owens [80] produced a review with a detailed list of chemicals, including Crystal Clean, EasyDECON DF200 (DF200) and trisodium phosphate (TSP) detergents that have been used to remediate methamphetamine contamination. TSP is often used as a Triple wash for low level, isolated contamination and it is labour intensive [81]. Sandia National Laboratories developed Crystal Clean and DF200 which were originally designed to be effective against a range of biological and chemical toxins [82]. Crystal Clean can be applied as a liquid, foam or fog, depending on which cleaning equipment is used [83]. In a study that assessed cleaning products for their efficacy in methamphetamine removal, it was reported that the hydrogen peroxide, quaternary ammonium and diacetin compounds provided a 100% reduction in methamphetamine [84]. Formula 409, quaternary ammonium compound and a hypochlorite solution called Chlorox Clean-up were also tested and resulted in 90%–95% and 57%–64% reductions, respectively. In another study, Owens [85] found that alkalised hydrogen peroxide removed >80% methamphetamine from the surface of painted dry wall and vinyl floor tiles; and the non-porous galvanised metal and glass surfaces. This was considered by Owens as a cost-effective cleaning product that is easily obtainable by the general public; however, precursor chemicals and by-products were not accounted for within the scope of this study. Therefore, this could be an indicator that the methamphetamine was degraded or transformed into another chemical form which would not result in a positive test [86,87]. Serrano et al. [74] evaluated various methods and chemicals for methamphetamine decontamination on clothing and building materials: drywall, plywood, sheet metal and glass. The results demonstrated that after three washing cycles, up to 99.9% methamphetamine could be removed from clothing. Four cleaning products were tested; sodium hypochlorite had the lowest percentage of removal (64%) after three washes, and when sampling different surfaces, sheet metal and glass had 100% removal with the all-purpose cleaner. The porous drywall and plywood had 80% removed from the surface after three washes with the all-purpose cleaner; however, it is important to note this does not account for any methamphetamine absorbed into the gyprock. Both surfaces were also encapsulated with latex (80%) and oil-based paint (100%). While encapsulation of methamphetamine is possible with oil-based paint, it has only been recommended for use after thorough remediation has been completed and has in no way been endorsed solely as a remediation technique [74].

While Owens [85] found a cheap, simple cleaning product was able to clean different surfaces, Serrano et al. [74] followed the cleaning with extraction quantification testing on the contaminated drywall. The results showed that only 40% of the methamphetamine was removed from the surface using the methanol wipes, and the remainder stayed on the dry wall. This study, in addition with research by Wright et al. [69], demonstrate the complexity of decontaminating methamphetamine, as it has been found to be re-released from porous surfaces into the environment over time [12]. Depending on the level of contamination in the premises, complete removal of building materials can be the safer, more cost-effective remediation option [85].

This is another area where more research is required. The current remediation techniques are mostly chemical-based strategies which require longitudinal studies to determine an accurate and complete efficacy result. As mentioned earlier, studies have demonstrated the difficulties in remediating methamphetamine and have shown to desorb over time, even after treatment [12].

## 5. Social and Ethical Considerations

Generally, homeowners or occupiers that suspect their house is contaminated with methamphetamine will hire an external investigator, such as an occupational hygienist, for an assessment and for sampling and testing of the premises. If methamphetamine contamination levels are higher than the relevant decontamination levels, then remediation is required. This often includes vacation of the property into temporary housing while treatment is being undertaken. Post-remediation testing is also necessary to ensure the cleaning was adequate and the property is safe for residents again. This means that those less able to absorb these costs are likely to be exposed to higher levels of methamphetamine and for longer periods compared with people that are financially stable and are able to pay for prompt testing, remediation and any necessary relocation. Methamphetamine remediation is very costly and the cost escalates with increasing contamination. Through the analysis of 1871 properties between 2012 and 2016, Tait et al. [88] estimated cost of remediation was 7000, 14,000 and 45,000 USD for small, medium and industrial sized laboratories respectively. Therefore, it has been suggested that home owners or landlords may choose not to have testing or remediation done because of the financial burden.

There is also the concern regarding the publicity methamphetamine contamination in properties has been receiving; the demand and popularity of remediation companies has greatly increased. Unfortunately, not all companies are honest; instead, some prey on the ill-informed and vulnerable customers. It is in the best interests of the company to find areas within the home with high levels of contamination to further their business [25]. All of these points highlight that methamphetamine contamination in properties is not only a public health issue but is also a major financial concern for those in a low socio-economic demographic.

## 6. Conclusions

Methamphetamine contamination within properties is a growing public health concern, particularly from former clandestine laboratories. There are currently research gaps in the areas of methamphetamine exposure levels and health effects, standard methods to determine contamination levels and remediation success. The body of research that is available often has conflicting conclusions, which serves to emphasise the complexity of the issue. Residues settle on surfaces; they can be absorbed [73], desorb over time [12], can have varied recovery rates [5] and can remain embedded in surfaces for years [69]. There are limited longitudinal studies that evaluate the long-term success of remediation treatments, and are highly varied recorded measurements for surface recovery, deposition and environmental samples. These variations create difficulties in the measurement of, estimated contamination of and exposure to methamphetamine. The lack of legislation and standardised methods creates diversity in methodology, mistrust in remediators from property owners and difficulties in maintaining consistency for law enforcement. There are also concerns relating to variation in training and the application of guidelines by regulators, commercial cleaning companies and homeowners. This is regardless of the state or country. The lack of conclusive current research supports the need for precautionary approach to be adopted. Further research to address these knowledge gaps and provide evidence for regulation to ensure public health protection is required.

## Figures and Tables

**Table 1:** A summary of methamphetamine decontamination limits from Australia, New Zealand and 23 states from the United States. This is not an exhaustive list of the US; information on all states can be found at Meth Lab Cleanup Company’s website [26].

Country	Acceptable Level of Methamphetamine (µg/100 cm^2^)	Title	Reference
Australia	0.5	Clandestine drug laboratory remediation guidelines	[16]
New Zealand	1.5	Testing and decontamination of methamphetamine contaminated properties	[27]
California	1.5	Technical support documents and fact sheets	[28]
Alaska	0.1	Guidance and standards for cleanup of illegal drug manufacturing sites	[29]
Arkansas	0.05	Clandestine laboratory remediation cleanup standards	[30]
Colorado	4.0 for limited exposure areas 1.5 for painted over surfaces	Cleanup of clandestine methamphetamine labs guidance document	[31]
Connecticut	0.1	Guidelines for the cleanup of Connecticut methamphetamine labs	[32]
Hawaii	0.1	Requirements for decontamination and cleanup of methamphetamine manufacturing sites	[33]
Idaho	0.1	Guidelines for cleaning up former methamphetamine labs	[34]
Indiana	0.5	Inspection and cleanup of property contaminated with chemicals used in the illegal manufacture of a controlled substance	[35]
Kansas	1.5	Cleaning up former methamphetamine labs	[36]
Kentucky	0.1	Kentucky cleanup guidance for methamphetamine contaminated properties	[37]
Michigan	0.5	Cleanup of clandestine drug laboratory guidance	[38]
Minnesota	0.1 for former labs 1.5 for use only	Clandestine drug lab general cleanup guidance	[39]
Montana	0.1	Methamphetamine contamination – Indoor property decontamination standards	[40]
Nebraska	0.1	Methamphetamine cleanup	[41]
New Hampshire	0.1	Guidance for the cleanup of clandestine chemical laboratories	[42]
New Mexico	1	Clandestine drug laboratory remediation	[43]
North Carolina	0.1	Illegal methamphetamine laboratory decontamination and re-occupancy guidelines	[44]
South Dakota	0.1	Guidelines for contamination reduction	[45]
Tennessee	0.1	Standards for testing and cleaning quarantined clandestine drug manufacturing sites	[46]
Utah	0.1	Illegal drug operations decontamination standards	[47]
Virginia	1.5	Guidelines for cleanup of residential property used to manufacture methamphetamine	[48]
Washington	1.5	Guidelines for environmental sampling at illegal drug manufacturing sites	[49]
Wyoming	1.5	Clandestine lab testing and remediation	[50]

## References

[1] McKetin R., McLaren J., Kelly E. (2005). The Sydney Methamphetamine Market: Patterns of Supply, Use, Personal Harms and Social Consequences.

[2] Darke S., Kaye S., McKetin R., Duflou J. (2008). Major physical and psychological harms of methamphetamine use. Drug Alcohol Rev..

[3] Goldberger B.A., Martin D.M., Graham N.A., Gold M.S. (2011). Second- and Third-hand Opium Smoke Exposure in Women and Children of Afghanistan. F1000Research.

[4] Martyny J.W., Arbuckle S.L., McCammon C.S., Erb N., Van Dyke M. (2008). Methamphetamine contamination on environmental surfaces caused by simulated smoking of methamphetamine. J. Chem. Health Saf..

[5] Bitter J.L. (2017). The persistence of illicit drug smoke residues and their recovery from common household surfaces. Drug Test. Anal..

[6] Al-Obaidi T.A., Fletcher S.M. (2014). Management of clandestine drug laboratories: Need for evidence-based environmental health policies. Environ. Health Prev. Med..

[7] Wright J., Edwards J., Walker S. (2016). Exposures associated with clandestine methamphetamine drug laboratories in Australia. Rev. Environ. Health.

[8] Cherney A., O’Reilly J., Grabosky P. (2006). Networks and Meta-regulation: Strategies Aimed at Governing Illicit Synthetic Drugs. Polic. Soc..

[9] Wright J., Kenneally M.E., Edwards J.W., Walker G.S. (2017). Adverse Health Effects Associated with Living in a Former Methamphetamine Drug Laboratory—Victoria, Australia, 2015. Morb. Mortal. Wkly. Rep..

[10] Thrasher D.L., Von Derau K., Burgess J. (2009). Health effects from reported exposure to methamphetamine labs: A poison center-based study. J. Med. Toxicol..

[11] Krieger J., Higgins D.L. (2002). Housing and Health: Time Again for Public Health Action. Am. J. Public Health.

[12] Poppendieck D., Morrison G., Corsi R. (2015). Desorption of a methamphetamine surrogate from wallboard under remediation conditions. Atmos. Environ..

[13] Institute of Environmental Science and Research Ltd (2016). Review of Remediation Standards for Clandestine Methamphetamine Laboratories: Risk Assessment Recommendations for a New Zealand Standard.

[14] Martyny J.W., Arbuckle S.L., McCammon C.S., Esswein E.J., Erb N., Van Dyke M. (2007). Chemical concentrations and contamination associated with clandestine methamphetamine laboratories. J. Chem. Health Saf..

[15] Barnes D.G., Dourson M. (1988). Reference dose (RfD): Description and use in health risk assessments. Regul. Toxicol. Pharmacol..

[16] Australian Crime Commission, Australian Government (2011). Clandestine Drug Laboratory Remediation Guidelines.

[17] The Environmental Health Standing Committee (enHealth) (2017). enHealth Guidance on: Clandestine Drug Laboratories and Public Health Risks.

[18] Government of South Australia (2011). South Australian Public Health Act.

[19] Victorian Government (2008). Public Health and Wellbeing Act.

[20] Government of New South Wales (2010). Public Health Act.

[21] Northern Territory Government (2011). Public and Environmental Health Act.

[22] Government of Western Australia (2016). Public Health Act.

[23] Queensland Government (2005). Public Health Act.

[24] Department of Health Clandestine Drug Laboratories—Are You at Risk?. https://healthywa.wa.gov.au/Articles/A_E/Clandestine-drug-laboratories-are-you-at-risk.

[25] Cohen H., Lewis D. Inside the Meth-Testing Industry Profiting from Families’ Fear. https://www.abc.net.au/news/2019-03-16/unregulated-meth-testing-industry-scamming-australian-public/10904806.

[26] Meth Lab Cleanup Company Meth Lab Cleanup Standards, Regulations & Disclosure. https://www.methlabcleanup.com/Meth-Lab-Cleanup-Standards.html.

[27] Standards New Zealand (2017). NZS 8510:2017 Testing and Decontamination of Methamphetamine Contaminated Properties.

[28] Department of Toxic Substances Control Illegal Drug Lab Removal Program. https://dtsc.ca.gov/erp/drug-lab-removals-erp/.

[29] Alaska Department of Environmental Conservation (2007). Guidance and Standards for Cleanup of Illegal Drug-Manufacturing Sites.

[30] Arkansas Department of Environmental Quality (2008). Clandestine Laboratory Remediation Cleanup Standards.

[31] Hazardous Materials and Waste Management Division (2003). Cleanup of Clandestine Methamphetamine Labs Guidance Document.

[32] Rusnak S.M., Ginsberg G., Toal B. (2006). Guidelines for the Cleanup of Connecticut Methamphetamine Labs.

[33] Department of Health (2010). Requirements for Decontamination and Cleanup of Methamphetamine Manufacturing Sites.

[34] Idaho Department of Health and Welfare (2006). Guidelines for Cleaning Up Former Methamphetamine Labs.

[35] Indiana Department of Environmental Management. Article 38 (2007). Inspection and Cleanup of Property Contaminated with Chemicals Used in the Illegal Manufacture of a Controlled Substance.

[36] Kansas Department of Health and Environment (2010). Cleaning Up Former Methamphetamine Labs.

[37] Energy & Environment Cabinet (2009). Kentucky Cleanup Guidance for Methamphetamine Contaminated Properties.

[38] Michigan Department of Community Health (2007). Cleanup of Clandestine Drug Laboratory Guidance.

[39] Minnesota Department of Health (2010). Clandestine Drug Lab General Cleanup Guidance.

[40] Montana Department of Environmental Quality (2005). Part 13. Methamphetamine Contamination—Indoor Property Decontamination Standards.

[41] Nebraska Department of Health and Human Services (2009). Chapter 24 Methamphetamine Cleanup.

[42] New Hampshire Department of Environmental Services (2007). Guidance for the Cleanup of Clandestine Chemical Laboratories.

[43] New Mexico Environment Department (2015). Part 5 Clandestine Drug Laboratory Remediation.

[44] Department of Health and Human Services (2005). Ilegal Methamphetamine Laboratory Decontamination and Re-occupancy Guidelines.

[45] State of South Dakota Guidelines for Contamination Reduction. https://www.methlabcleanup.com/SD%20REGULATIONS.pdf.

[46] Tennessee Department of Environment and Conservation (2012). Standards for Testing and Cleaning Quarantined Clandestine Drug Manufacturing Sites.

[47] State of Utah (2015). Illegal Drug Operations Decontamination Standards.

[48] Virginia Department of Health (2013). Guidelines for Cleanup of Residential Property Used to Manufacture Methamphetamine.

[49] State of Washington (2015). Decontamination of Illegal Drug Manufacturing or Storage Sites.

[50] State Emergency Response Commission Clandestine Lab Testing and Remediation.

[51] Ministry of Health (2010). Guidelines for the Remediation of Clandestine Methamphetamine Laboratory Sites.

[52] Housing New Zealand Corporation (2018). Methamphetamine Contamination Housing New Zealand’s Response.

[53] New Zealand Government (1956). Health Act.

[54] New Zealand Government (2004). Building Act.

[55] Russell M., McKinnel M., Ivory B. (2018). Methamphetamine Contamination.

[56] Bardsley A., Low F. (2018). Methamphetamine Contamination in Residential Properties: Exposures, Risk Levels and Interpretation of Standards.

[57] Salocks C.B. (2009). Assessment of Children’s Exposure to Surface Methamphetamine Residues in Former Clandestine Methamphetamine Labs, and Identification of a Risk-Based Cleanup Standard for Surface Methamphetamine Contamination.

[58] Salocks C., Golub M., Kaufman F. (2009). Development of a Reference Dose (RfD) for Methamphetamine.

[59] Colorado Department of Public Health and Environment (2005). Support for Selection of a Cleanup Level for Methamphetamine at Clandestine Drug Laboratories.

[60] Aldhous P. (2008). Unsafe house. New Sci..

[61] Levine S.L. (2004). Poison in Our Own Backyards: What Minnesota Legislators Are Doing to Warn Property Purchasers of the Dangers of Former Clandestine Methamphetamine Labs Other Recent Developments in Minnesota Law: Note. William Mitchell Law Rev..

[62] Krause E.I. (2006). Take My Property Please—Who Should Bear the Burden of Cleaning up Toxic Methamphetamine Lab Waste Comment. Cathol. Univ. Law Rev..

[63] Tenancy Tribunal Properties Affected by Meth. https://www.tenancy.govt.nz/starting-a-tenancy/renting-affected-properties/renting-a-property-affected-by-methamphetamine-p/.

[64] Baker M.G., Zhang J., Blakely T., Crane J., Saville-Smith K., Howden-Chapman P. (2016). Collaborating with a social housing provider supports a large cohort study of the health effects of housing conditions. BMC Public Health.

[65] Marmot M., Allen J., Bell R., Bloomer E., Goldblatt P. (2012). WHO European review of social determinants of health and the health divide. Lancet.

[66] United States Government (2007). Methamphetamine Remediation Research Act.

[67] United States Environmental Protection Agency (2013). Voluntary Guidelines for Methamphetamine Laboratory Cleanup.

[68] Van Dyke M., Martyny J.W., Serrano K.A. (2014). Methamphetamine Residue Dermal Transfer Efficiencies from Household Surfaces. J. Occup. Environ. Hyg..

[69] Wright J., Walker G.S., Ross K.E. (2019). Contamination of Homes with Methamphetamine: Is Wipe Sampling Adequate to Determine Risk?. Int. J. Environ. Res. Public Health.

[70] Van Dyke M., Erb N., Arbuckle S., Martyny J. (2009). A 24-Hour Study to Investigate Persistent Chemical Exposures Associated with Clandestine Methamphetamine Laboratories. J. Occup. Environ. Hyg..

[71] Harris D.S., Boxenbaum H., Everhart E.T., Sequeira G., Mendelson J.E., Jones R.T. (2003). The bioavailability of intranasal and smoked methamphetamine. Clin. Pharmacol. Ther..

[72] Madireddy S.B., Bodeddula V.R., Mansani S.K., Wells M.J., Boles J.O. (2013). Wipe sampling of amphetamine-type stimulants and recreational drugs on selected household surfaces with analysis by ultra-performance liquid chromatography quadrupole time-of-flight mass spectrometry. J. Hazard. Mater..

[73] Abdullah A.F., Miskelly G.M. (2010). Recoveries of trace pseudoephedrine and methamphetamine residues from impermeable household surfaces: Implications for sampling methods used during remediation of clandestine methamphetamine laboratories. Talanta.

[74] Serrano K.A., Martyny J.W., Kofford S., Contreras J.R., Van Dyke M.V. (2012). Decontamination of clothing and building materials associated with the clandestine production of methamphetamine. J. Occup. Environ. Hyg..

[75] Salocks C.B., Hui X., Lamel S., Qiao P., Sanborn J.R., Maibach H.I. (2012). Dermal exposure to methamphetamine hydrochloride contaminated residential surfaces: Surface pH values, volatility, and in vitro human skin. Food Chem. Toxicol..

[76] Patrick G., Daniell W., Treser C. (2009). Residual methamphetamine in decontaminated clandestine drug laboratories. J. Occup. Environ. Hyg..

[77] Morrison G., Shakila N.V., Parker K. (2015). Accumulation of gas-phase methamphetamine on clothing, toy fabrics, and skin oil. Indoor Air.

[78] Parker K., Morrison G. (2016). Methamphetamine absorption by skin lipids: Accumulated mass, partition coefficients, and the influence of fatty acids. Indoor Air.

[79] Brewer K., Shults T.F., Machin J., Kudrimoti S., Eisenberg R.L., Hartman P., Wang C., Fenger C., Beaumier P., Tobin T. (2016). A cluster of trace-concentration methamphetamine identifications in racehorses associated with a methamphetamine-contaminated horse trailer: A report and analysis. Can. Vet. J..

[80] Owens C.V. (2017). Remediation of manufactured methamphetamine in clandestine laboratories. A literature review. J. Chem. Health Saf..

[81] DeconASSIST What Methods are Considered Best Practice for Methamphetamine Decontamination?. https://deconassist.com.au/blogs/news/what-methods-are-considered-best-practice-for-methamphetamine-decontamination.

[82] Intelagard EasyDECON DF200. https://intelagard.com/portfolio/easydecon-df200/.

[83] Intelagard Crystal Clean. https://intelagard.com/portfolio/crystal-clean/.

[84] Martyny J.W. Oxidizer Decontamination of Building Materials Contaminated with Methamphetamine. http://enviro-decon.com/files/2014/03/Final-Oxidizer-decontamination-Study-1.pdf.

[85] Owens C.V. (2017). The efficacy of alkalized liquid hydrogen peroxide for the remediation of manufactured methamphetamine in clandestine laboratories (vol 24, pg 2, 2017). J. Chem. Health Saf..

[86] Hernandez F., Castiglioni S., Covaci A., de Voogt P., Emke E., Kasprzyk-Hordern B., Ort C., Reid M., Sancho J.V., Thomas K.V. (2018). Mass spectrometric strategies for the investigation of biomarkers of illicit drug use in wastewater. Mass Spectrom. Rev..

[87] Rindelaub J.D., Miskelly G.M. (2019). Potential for the Remediation of Methamphetamine Contamination Using Ozone. J. Forensic Sci..

[88] Tait R.J., Allsop S., Cartwright K., Ferrante A., Gray D., Kaye S., McKetin R., Pidd K., Ritter A., Roche A. Remediation and property values: The costs of clandestine methamphetamine manufacuring in Australia. Proceedings of the APSAD Scientific Alcohol and Drug Conference.

